# Evaluation of real-time nutrient analysis of fertilized raspberry using petiole sap

**DOI:** 10.3389/fpls.2022.918021

**Published:** 2022-08-05

**Authors:** Qianwen Lu, Carol Miles, Haiying Tao, Lisa DeVetter

**Affiliations:** ^1^Department of Plant Science and Landscape Architecture, University of Connecticut, Storrs, CT, United States; ^2^Northwestern Washington Research and Extension Center, Washington State University, Mount Vernon, WA, United States

**Keywords:** *Rubus idaeus* L., nitrogen fertilizer rate, leaf tissue, nutrient management, compact ion meter, correlation

## Abstract

The time delay in receiving conventional tissue nutrient analysis results caused red raspberry (*Rubus idaeus* L.) growers to be interested in rapid sap tests to provide real-time results to guide immediate nutrient management practices. However, sap analysis has never been conducted in raspberry. The present work aimed to evaluate the relationship of petiole sap nitrate (NO_3_^–^), potassium (K^+^), and calcium (Ca^2+^) concentrations measured using compact ion meters and leaf tissue total nitrogen (TN), potassium (K), and calcium (Ca) concentrations measured using conventional tissue nutrient analysis. The relationship of petiole sap NO_3_^–^ and leaf tissue TN concentrations with plant growth and production variables was also explored. Fertilizer treatments of urea were surface applied to raised beds of established “Meeker” floricane red raspberry plots at control, low, medium, and high rates (0, 34, 67, and 101 kg N ha^–1^, respectively) in 2019 and 2020. The experiment was arranged in a randomized complete block design with three replications. Whole leaves were collected from representative primocanes in mid- and late- July and August 2019 and 2020 (i.e., four sampling time points per year). At each sampling time point, a subsample of leaves was used for petiole sap analyses of NO_3_^–^, K^+^, and Ca^2+^ concentrations using compact ion meters, and conventional tissue testing of leaf tissue TN, K, and Ca concentrations, respectively. There were no interactions between N fertilizer rate and year nor between N fertilizer rate and sampling time. No significant differences were found due to N fertilizer rate for petiole sap NO_3_^–^, K^+^, Ca^2+^ nor leaf tissue TN, K, Ca concentrations. However, significant year and sampling time effects occurred in measured petiole sap and leaf tissue nutrient concentrations. Overall, the correlations between petiole sap NO_3_^–^ and leaf tissue TN, petiole sap Ca^2+^ and leaf tissue Ca, petiole sap K^+^ and leaf tissue K concentrations were non-strong and inconsistent. Future research is warranted as the interpretation of correlations between raspberry petiole sap and leaf tissue nutrient concentrations were inconclusive.

## Introduction

Washington State leads processed red raspberry (*Rubus idaeus* L.) production in the United States of America (USA) with a 3-year average of approximately 28,470 tons of fruit produced mostly in the northwest of the state in 2019–2021. Most of the raspberry production in Washington is from the floricane type and had an average annual value of $70.7 million in 2019–2021 (USDA NASS, 2022). In order to maintain the production of this high-value perennial crop, fertilizers are applied annually based on plant nutrient analyses and observations of cane growth (Hart et al., 2006). Conventional plant tissue nutrient analysis, which uses dried and ground tissue samples, is the standard plant nutrient testing method (Hart et al., 2006; Carson et al., 2016). However, this testing method requires time for commercial laboratory analysis as samples are dried, ground, digested or extracted, and then analyzed (Miller et al., 2013). The time from sample collection to receipt of the results by the grower can be up to 2 weeks. This time delay has led to the interest of some growers and consultants in trying quick plant nutrient tests to provide real-time results for nutrient management.

Sap analysis using compact ion meters is one such rapid plant nutrient test, which analyses plant xylem and phloem sap plus apoplastic, cytosolic, and vacuolar solutions (Farneselli et al., 2014; Taiz et al., 2015; Llanderal et al., 2020). Only the specific ionic form of a nutrient can be measured through sap analysis using a compact ion meter, which is different than conventional tissue nutrient analysis that measures the concentration of a given nutrient in all forms (Rundle, 2011). Sap analysis using compact ion meters may be utilized in combination with conventional tissue nutrient analysis to evaluate plant nutrient status and help growers and crop consultants make nutrient management decisions. Some studies have stated that sap analyses are cheaper and faster than conventional tissue nutrient analyses (Nagarajah, 1999; Gangaiah et al., 2016; Rodríguez et al., 2021). However, no studies show detailed cost comparisons between sap analysis and conventional tissue nutrient analysis.

Alternative nutrient analysis methods, such as rapid petiole sap analysis, are often compared to the standard conventional tissue nutrient analysis method to test its potential utility. Some studies have correlated sap to conventional tissue nutrient analysis results to test the sap analysis for assessing plant nutrient status. In annual crops, Locascio et al. (1997) found that correlation coefficients (r) were between −0.2 and 0.8 for the relationship between petiole sap nitrate nitrogen (NO_3_-N) and leaf tissue total nitrogen (TN) concentrations in tomato (*Solanum lycopersicum* L.) grown for 4, 6, 8, 10, and 12 weeks after transplanting at two different locations in Florida, United States, with the highest r ranging 0.6–0.8 for 6–8 weeks after transplanting. Another study carried out in Florida, United States reported a non-significant correlation between tomato petiole sap NO_3_-N and leaf tissue TN concentration 14 weeks after transplanting (Carson et al., 2016). Tomato undergoing rapid vegetative growth and first flowering 6–8 weeks after transplanting (Shamshiri et al., 2016) utilizes NO_3_-N mainly to support vegetative growth of new tissue, which may result in relatively strong correlations between petiole sap NO_3_-N and leaf tissue TN concentrations. While at other growth stages that coincide with early growth, first, second, and third harvest (4, 10, 12, and 14 weeks after transplanting, respectively; Carson et al., 2016; Shamshiri et al., 2016), when NO_3_-N is mainly used to support seedling growth and fruiting (Taiz et al., 2015). At these developmental stages, most of the NO_3_-N will not be assimilated or stored in leaf tissues, leading to poor correlations between petiole sap NO_3_-N and leaf tissue TN concentrations. Majić et al. (2008) reported a significant relationship (0.41 < *r* < 0.80) between petiole sap NO_3_-N and leaf tissue TN concentrations for potato (*Solanum tuberosum* L. cv. Adora, Cleopatra, and Liseta) grown for 65, 75, 85, and 95 days after planting in Bosnia and Herzegovina with the highest *r* = 0.80 at 65 days after planting, which corresponds with the growth stage of early tuber initiation. During this growth stage, rapid nutrient uptake occurs, and nutrients are mainly translocated or stored in vegetative leaf tissues (Jia et al., 2018), thus, a strong correlation between petiole sap NO_3_-N and leaf tissue TN concentrations is expected. After tuber initiation, nutrients in potatoes are mainly translocated to flowers and tubers (Taiz et al., 2015). Therefore, correlations between petiole sap NO_3_-N and leaf tissue TN concentrations at these later developmental stages may be poor. Zhou and Yin (2018) found the relationship between petiole sap NO_3_^–^ and leaf tissue TN concentrations for cotton (*Gossypium hirsutum* L.) cultivated in west Tennessee, United States varied by growth stage and the coefficients of determination (R^2^) were between 0.42 and 0.71 with *R*^2^ = 0.71 observed at mid-bloom. A case study in Inner Mongolia, China reported a strong relationship (*r* = 0.92) between potato (cv. Kexin-1) petiole sap potassium (K^+^) and shoot tissue potassium (defined as K hereafter) concentrations across all sampling dates from 30 to 75 days after emergence during the vegetative growth stage (Shi et al., 2019). Gangaiah et al. (2016) also reported a strong correlation (0.80 < *r* < 0.99) between petiole sap K^+^ and leaf tissue K concentrations in the vegetative growth stage of pak choi (*Brassica rapa* L. var. Chinensis group “Bonsai”) planted in a greenhouse for 5 weeks in Hawaii, United States.

In perennial systems, Pino et al. (2012) reported a positive correlation between 1-year-old grapevine (*Vitis vinifera* L. cv. Red Globe) petiole sap NO_3_^–^ concentration and plant N content in leaf blades and roots with *R*^2^ = 0.74 at full bloom. In apple (*Malus domestica* Borkh cv. Red Gala), petiole sap Ca^2+^ concentration correlated well with leaf tissue Ca concentration 28–147 days after full bloom with *R*^2^ = 0.83 (Almeida et al., 2020). Nagarajah (1999) found a positive correlation between grape (cv. Sultana) petiole sap K^+^ and total petiole K concentrations during bloom with *R*^2^ = 0.86. These previous findings suggest that the relationship between sap and conventional tissue nutrient analysis is variable in crops and depends on the developmental growth stage. Sap analysis using compact ion meters has not been performed in many perennial crops, including floricane red raspberry, which is a unique perennial fruit crop with biennial canes that are vegetative in the first year of growth and fruiting the following year before senescing. Tissue nutrient concentrations vary dynamically in this unique growing system. It has been reported that the floricane red raspberry mainly utilizes fertilizer and soil N as well as stored N for vegetative and reproductive growth, respectively. Absorbed nutrients translocate from senesced, fruiting floricanes to root, crown, and over-wintering vegetative primocanes postharvest to fuel plant growth in the following Spring (Rempel et al., 2004). The objective of this research was to evaluate the relationship of petiole sap nutrient concentrations measured using ion selective electrode compact meters with leaf tissue nutrient concentrations measured with conventional tissue nutrient analysis methods in red raspberry fertilized at different N rates. The results of this study will provide information to researchers, crop consultants, and growers to better understand the application of rapid petiole sap analysis using ion selective electrode compact meters to measure floricane red raspberry nutrient status for real-time decision making.

## Materials and methods

### Experimental site

**FIGURE 1:**
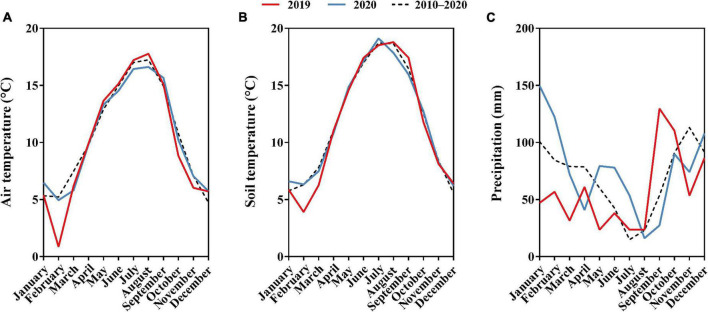
**(A)** Monthly average air temperature (°C) of 2019, 2020, and 2010–2020. **(B)** Monthly average soil temperature (°C) of 2019, 2020, and 2010–2020. **(C)** Monthly average precipitation (mm) of 2019, 2020, and 2010–2020 in the experimental area located in northwestern Washington. Data provided by WSU AgWeatherNet.

The experiment was carried out in an established field of “Meeker” floricane red raspberry located at the Washington State University Northwestern Washington Research and Extension Center (WSU NWREC) in Mount Vernon, WA, United States (48° 44′20′′ N, 122° 39′16′′ W) in 2019 and 2020. The 0.03 ha (37 m in length × 9 m in width) field was planted with raspberry on raised beds (0.6 m wide at the top and 0.25 m tall) in May 2017. In-row plant spacing was 60 cm and between-row spacing was 3 m. The soil type of the site is a silt loam, characterized as a fine-silty, mixed, superactive, non-acid, mesic Fluvaquentic Endoaquepts (United States Department of Agriculture [USDA], 2021). The characteristics of the raspberry field soil are shown in Table 1. The site was managed using standard commercial raspberry production practices for the region (DeVetter et al., 2022). Average monthly air temperature, soil temperature at a depth of 20 cm, and precipitation of the experimental area in 2019, 2020, and 2010–2020 are shown in Figure 1. Total precipitation was 685 and 907 mm for 2019 and 2020, respectively (Washington State University AgWeatherNet, 2021).

**TABLE 1:** Initial soil characteristics of a “Meeker” floricane red raspberry field in northwestern Washington (48° 44′20′′ N, 122° 39′16′′ W).

pH^z^	Cation exchange capacity (meq/100 g)	Organic matter (%)	ENR^y^ (kg N ha^–^^1^)	ppm											
				NO_3_-N	P^x^	K	Ca	Mg	SO_4_-S	Na	Mn	B	Fe	Cu	Zn
6.4	6.7	2.8	96	5	100	202	930	103	3	11	2.0	0.3	69	3.0	1.8

Soil was sampled in March 2019 with a standard 2.5 cm soil probe to a 30 cm depth.

^z^Measured using soil:H_2_O at a 1:1 ratio.

^y^Estimated nitrogen release (ENR) was estimated based on the percentage of organic matter in the soil.

^x^Bray I P.

### Experimental design

The experimental design was a randomized complete block with four treatments replicated three times. The experimental unit was a plot that contained 11–14 raspberry plants with 8–15 canes per plant. Treatment was fertilizer rate at low, medium, and high rates (34, 67, and 101 kg N ha^–1^, respectively) plus a no fertilizer control (0 kg N ha^–1^) applied using granular urea (46N-0P-0K). Half of the fertilizer was applied in mid-April (approximately a week before primocane emergence) and the other half in late-May (approximately a month before first harvest) in both 2019 and 2020, which was in accordance with the regional nutrient management guide (Hart et al., 2006). During fertilizer application, urea was uniformly sprinkled over the surface of the raised bed. Plants were drip irrigated for at least 2 h right after fertilization in order to incorporate urea into the soil and reduce volatilization. No additional fertilizer was applied beyond the fertilizer treatments. Within each year, drip irrigation is incurred every 2–4 days from June to August for 2 h with the dripper flow rate of 1.52 L h^–1^ at each irrigation event.

### Plant and soil sample collection

In each plot, the fourth most recent, fully expanded whole leaves (approximately 30 cm from the tip) were collected from 30 representative primocanes free of disease and damage according to Bundt et al. (1997); Carson et al. (2016), and Shi et al. (2019). Sampling occurred from mid-July to late August at a 14-day interval such that there were four sample collections (mid- and late- July and August) in both 2019 and 2020. Sample collection and timing were in accordance with the protocol outlined by Hart et al. (2006) who reported that raspberry nutrient concentrations are most stable during the proposed sampling time in the Pacific Northwest region. During each sampling time, leaf samples were collected between 09:00 and 14:00 h on sunny days, when the diurnal variation of petiole sap NO_3_^–^, K^+^, and Ca^2+^ concentrations was reported to be minimal (Herdel et al., 2001). Leaf samples were placed in paper bags, immediately transported to the laboratory located at WSU NWREC, and prepared for further analysis right after collection.

Baseline soil sampling was done on 22 March 2019. Soil samples consisting of 30 cores were randomly selected from the area 5 m around the experimental field using a standard 2.5 cm wide soil probe to a 30 cm depth. Soil from all cores was combined and mixed thoroughly as one composite sample. Post-harvest comprehensive soil sampling was done on 15 September 2020. Soil samples consisting of 12 cores per plot, with four cores on each side plus four cores in the center of the raised bed in the row, were collected using a standard 2.5 cm wide soil probe to a 30 cm depth. Cores from the same plot were combined and mixed thoroughly as one composite sample.

### Soil characteristic analysis

Field-moist soil samples were sent to a commercial laboratory (Brookside Laboratories, Inc., New Bremen, OH, United States) within 24 h after sampling for soil analysis. Soil organic matter was measured using the loss of ignition method at 360°C for 12 h (Schulte and Hopkins, 1996). Soil estimated nitrogen release was a computed estimate of nitrogen that may be released annually through organic matter decomposition. It was estimated based on the percentage of organic matter in the soil. Soil K and Ca were extracted using the Mehlich III extraction method (Mehlich, 1984) and then tested with an inductively coupled plasma spectrometer (Thermo Scientific ICAP 7000 series; Thermo Instruments, Waltham, MA, United States).

### Plant petiole sap and leaf tissue nutrient analysis

On each sampling date, 25 of the 30 sampled leaves per plot were prepared for petiole sap analysis by removing the leaflets so that only the petiole remained. Petioles were cut into small, ∼1 cm sections with a hand-held pruner. Petiole sections were placed into a plant press (Zyliss, Farnborough, United Kingdom), manually pressed for approximately 10 s using consistent pressure, and approximately 20 drops of sap were collected into a 5 ml beaker. The sap was not diluted and followed approaches outlined in previous studies (Folegatti et al., 2005; Altamimi et al., 2013; Carson et al., 2016). Extracted sap was analyzed for NO_3_^–^, K^+^, and Ca^2+^ concentrations using compact ion meters with ion selective electrode sensors (Horiba LAQUAtwin; Spectrum Technologies, Aurora, IL, United States). Meters were calibrated before use and recalibrated after every 10 measurements utilizing a standard solution provided by the manufacturer (Altamimi et al., 2013). There were four analytical replications per sample of 25 leaves. After each measurement, the sensor was rinsed with deionized water and blotted clean (Kimtech; Kimberly-Clark Inc., Kent, WA, United States). Each sap measurement was done within 1–2 min after extraction to ensure consistency among samples, and all measurements were completed within 5 h after sample collection.

The remaining five leaves plus petioles were kept intact, dried at 38°C for 5 days until constant weight, and then sent to a commercial laboratory (Brookside Laboratories, Inc., New Bremen, OH, United States) for plant tissue macro- and micro-nutrient analysis. Leaf tissue TN concentration was measured by the combustion method (Elementar EL Cube C/N combustion analyzer, Elementar Inc., Langenselbold, Germany). Leaf tissue K and Ca concentrations were measured using the nitric acid and hydrogen peroxide digestion method and then tested with inductively coupled plasma mass spectrometry (Thermo 6500 Duo; Thermo Instruments, Waltham, MA, United States) (Miller et al., 2013).

### Primocane height and number

Primocane height and number were recorded from three representative plants within the interior of each plot on 15 September 2020. Primocane height was determined by measuring the height of the tallest primocane per plant, starting at the base of the crown and extending to the tallest leaf tip. Primocane number was determined by counting the number of primocanes emerging from the base of the crown per plant.

### Fruit yield

Raspberry fruit was machine harvested (Littau, # R0012; Littau Harvester Inc., Lynden, WA, United States) by plot every 3–4 days for a total of 10 times from 29 June to 4 August 2020. No yield data were collected in 2019 due to harvester malfunction. The total yield per plant across the whole harvest season was calculated.

### Costs of plant nutrient tests

Cost comparison for the two plant nutrient testing methods was carried out based on the recommended number of samples that should be collected for each method. Costs for collection and analysis were estimated for each test method based on current labor costs, laboratory testing fees, and costs of supplies. The cost of the two methods was then estimated and compared for the 6-year expected life span of a red raspberry planting in Washington.

### Statistical analyses

All data were subjected to analysis of variance with linear mixed-effects models using the lme() function in the nlme package built in R (R version 3.6.3; Boston, MA, United States). Block was always treated as a random effect. N fertilizer rate, year, and sampling time were fixed effects. The assumptions of normality and homogeneity of variance were checked by visual inspection of residual plots. Soil K data were sine transformed to improve the normality and homogeneity of the residuals. All means are reported in original units.

Both petiole sap and dried leaf tissue nutrient data were first analyzed using two-way factor analysis with N fertilizer rate and year as the factors with repeated measures for the 2-year data. Then, data within each year were analyzed using two-way factor analysis with N fertilizer rate and sampling time as the factors. Lastly, data within each sampling time point were analyzed using one-way factor analysis with N fertilizer rate as the factor. Given measured soil variables, primocane height and number, and yield data were only collected in 2020, these data were analyzed using one-way factor analysis with N fertilizer rate as the factor. A Tukey’s honestly significant difference test in the multcomp package was used for *post hoc* comparisons at the 5% level of significance to compare treatment means.

Additionally, petiole sap NO_3_^–^ and leaf tissue TN, petiole sap K^+^ and leaf tissue K, and petiole sap Ca^2+^ and leaf tissue Ca concentrations were subjected to Pearson correlation and linear regression analyses to determine the direction and strength of the relationship. Exponential, logarithmic, polynomial, and power relationships were also considered before deciding to use linear regression. An autocorrelation ACF() function in the nlme package was also performed with the temporal series of petiole sap NO_3_^–^, K^+^, Ca^2+^ and leaf tissue TN, K, and Ca concentrations. The autocorrelation function is a way to monitor the effectiveness of the regression model to capture the time trend when the data have been collected over time.

## Results

### Petiole sap and leaf tissue nutrient concentration

For each petiole sap and leaf tissue nutrient, there were no significant effects due to the N fertilizer rate within each sampling time, within each year, nor across the 2-year study; there was also no year-by-treatment interactions nor sampling time by treatment interactions (Supplementary Tables 1, 3, 4). A year effect was found for petiole sap K^+^ and leaf tissue K and Ca concentrations with petiole K^+^ and leaf tissue Ca concentrations greater in 2019 than in 2020 whereas leaf tissue K concentrations were greater in 2020 than in 2019 (Supplementary Tables 1, 2). Sampling time effects were found for all measured petiole sap and leaf tissue nutrient concentrations (Tables 2–4).

**TABLE 2:** “Meeker” floricane red raspberry petiole sap nitrate (NO_3_^–^) and leaf tissue total nitrogen (TN) concentrations by nitrogen fertilizer rate treatments and sampling time, 2019 and 2020.

Treatment	Petiole sap NO_3_^–^ (ppm)		Leaf tissue TN (%)	
	2019	2020	2019	2020
Control (0 kg N ha^–1^)	1497.7 ± 200.0^z^	1873.3 ± 203.0	2.82 ± 0.11	2.97 ± 0.07
Low (34 kg N ha^–1^)	1681.5 ± 196.1	1843.8 ± 132.3	2.86 ± 0.08	2.93 ± 0.05
Medium (67 kg N ha^–1^)	1882.7 ± 193.0	1812.5 ± 218.0	2.97 ± 0.11	2.96 ± 0.06
High (101 kg N ha^–1^)	1906.3 ± 236.2	1708.3 ± 134.0	2.77 ± 0.05	2.87 ± 0.04
*P*-value	0.68	0.94	0.47	0.79

**Sampling time**	**Petiole sap NO_3_^–^ (ppm)**		**Leaf tissue TN (%)**	
	**2019**	**2020**	**2019**	**2020**

Mid-July	1251.9 ± 119.2 b	2372.9 ± 176.4a	3.01 ± 0.10a	2.89 ± 0.06
Late-July	2300.0 ± 166.7a	2089.6 ± 115.6a	2.97 ± 0.10a	2.86 ± 0.04
Mid-August	2131.3 ± 198.6a	1407.1 ± 93.6b	2.67 ± 0.06b	3.04 ± 0.07
Late-August	1285.0 ± 131.1b	1368.3 ± 62.9b	2.78 ± 0.07ab	2.95 ± 0.04
*P*-value	<0.0001	<0.0001	0.003	0.09

Data are displayed by year given raspberry is a perennial crop.

^z^Data are displayed as means ± SE (standard error) (*n* = 12); means followed by different letter within a column are significantly different at *P* ≤ 0.05 using a means comparison with a Tukey’s honestly significant difference test.

**TABLE 3:** “Meeker” floricane red raspberry petiole sap potassium (K^+^) and leaf tissue K concentrations by sampling time, 2019 and 2020.

Sampling time	Petiole sap K^+^ (ppm)		Leaf tissue K (%)	
	2019	2020	2019	2020
Mid-July	4068.8 ± 154.0c^z^	4900.0 ± 121.7a	1.86 ± 0.07a	2.06 ± 0.05a
Late-July	4420.8 ± 129.8c	3872.9 ± 80.6c	1.64 ± 0.07ab	1.83 ± 0.06b
Mid-August	5485.4 ± 136.8a	4441.7 ± 107.5b	1.47 ± 0.08b	1.78 ± 0.03b
Late-August	4979.2 ± 90.4b	3614.6 ± 97.3c	1.51 ± 0.04b	1.79 ± 0.05b
*P*-value	<0.0001	<0.0001	0.0007	0.001

Data are displayed by year given raspberry is a perennial crop.

^z^Data are displayed as means ± standard error (*n* = 12); means followed by different letter within a column are significantly different at *P* ≤ 0.05 using a means comparison with a Tukey’s honestly significant difference test.

**TABLE 4:** “Meeker” floricane red raspberry petiole sap calcium (Ca^2+^) and leaf tissue Ca concentrations by sampling time, 2019 and 2020.

Sampling time	Petiole sap Ca^2+^ (ppm)		Leaf tissue Ca (%)	
	2019	2020	2019	2020
Mid-July	79.8 ± 7.2ab^z^	80.4 ± 6.7a	0.63 ± 0.01b	0.66 ± 0.02a
Late-July	58.8 ± 1.8c	79.7 ± 2.5ab	0.68 ± 0.01a	0.62 ± 0.01ab
Mid-August	98.5 ± 7.7a	64.2 ± 1.9bc	0.67 ± 0.01a	0.57 ± 0.02b
Late-August	77.0 ± 3.4bc	63.4 ± 2.6c	0.61 ± 0.01b	0.60 ± 0.01ab
*P*-value	0.0003	0.006	0.0001	0.008

Data are displayed by year given raspberry is a perennial crop.

^z^Data are displayed as means ± standard error (*n* = 12); means followed by different letter within a column are significantly different at *P* ≤ 0.05 using a means comparison with a Tukey’s honestly significant difference test.

### Relationship between petiole sap and leaf tissue nutrient concentration

**FIGURE 2:**
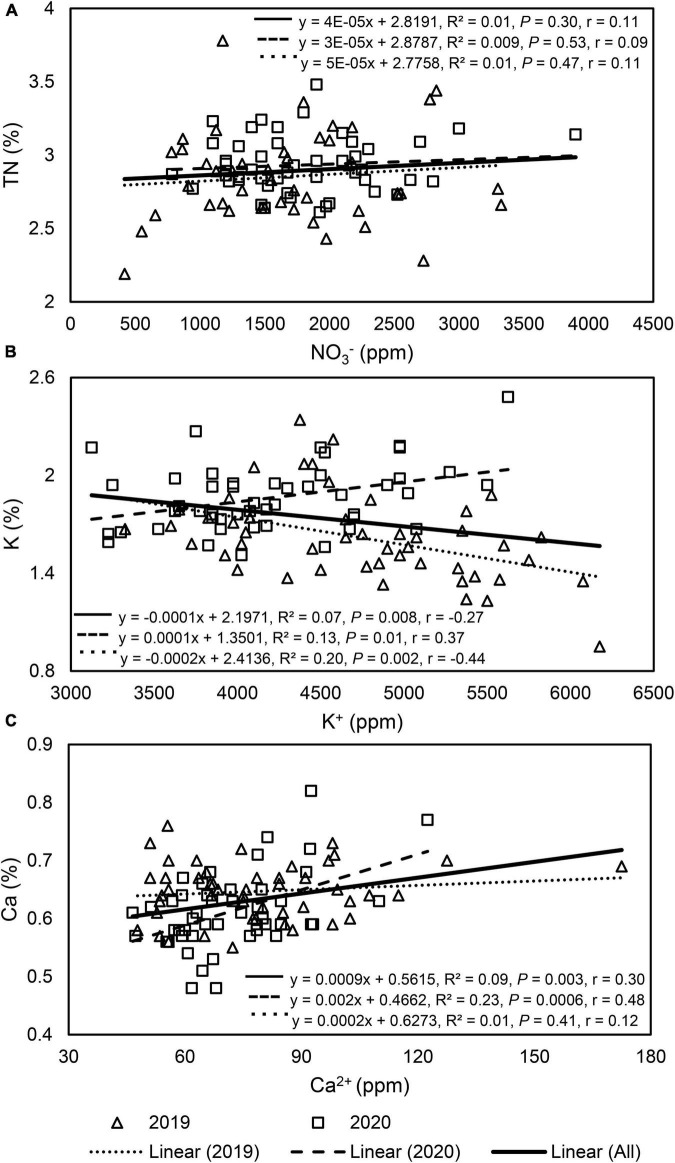
Linear regression graphs, equations, coefficients of determination (R^2^), *P*-values, and Pearson correlation coefficients (r) of each paired variable in “Meeker” floricane raspberry: **(A)** petiole sap nitrate (NO_3_^–^) and leaf tissue total nitrogen (TN), **(B)** petiole sap potassium (K^+^) and leaf tissue K, and **(C)** petiole sap calcium (Ca^2+^) and leaf tissue Ca. In each graph, the dashed and dotted line was each analyzed with 48 paired samples by year, while the solid line was analyzed with all (*n* = 96) paired samples throughout 2 years.

The relationship between petiole sap NO_3_^–^ and leaf tissue TN concentrations was insignificant when pooled across 2 years and when analyzed by year (Figure 2). Within each year, the relationship between petiole sap NO_3_^–^ and leaf tissue TN concentrations varied by N fertilizer rate and sampling time. In 2019, petiole sap NO_3_^–^ concentration was strongly positively correlated to leaf tissue TN concentration in unfertilized (0 kg N ha^–1^) plants, and 76% of the variation of leaf tissue TN concentration was explained by petiole sap NO_3_^–^ concentration. Petiole sap NO_3_^–^ concentration was also positively correlated to leaf tissue TN concentration in late-August 2019 and 32% of the variation in leaf tissue TN concentration could be explained by petiole sap NO_3_^–^ concentration. All other relationships between petiole sap NO_3_^–^ and leaf tissue TN concentration for other N fertilizer rates and sampling times in 2019 were insignificant. In 2020, petiole sap NO_3_^–^ concentration was positively correlated to leaf tissue TN concentration only in mid-July and 34% of the variation in leaf tissue TN concentration could be explained by the petiole sap NO_3_^–^ concentration (Table 5).

**TABLE 5:** Pearson correlation coefficients (r), linear regression coefficients of determination (R^2^), and the *P*-values of paired variables by nitrogen fertilizer rate treatments in 2019 and 2020.

Paired variable	Treatment	2019			2020		
		Correlation coefficient (r)	Coefficient of determination (R^2^)	*P*-value	Correlation coefficient (r)	Coefficient of determination (R^2^)	*P*-value
Petiole sap NO_3_^–^ and leaf tissue TN	Control (0 kg N ha^–1^)	0.87	0.76	0.0002	0.32	0.10	0.31
	Low (34 kg N ha^–1^)	0.08	0.01	0.80	−0.30	0.09	0.35
	Medium (67 kg N ha^–1^)	−0.39	0.15	0.21	0.21	0.04	0.52
	High (101 kg N ha^–1^)	−0.31	0.10	0.33	−0.37	0.14	0.23

**Paired variable**	**Sampling time**	**2019**			**2020**		
		**Correlation coefficient (r)**	**Coefficient of determination (R^2^)**	***P*-value**	**Correlation coefficient (r)**	**Coefficient of determination (R^2^)**	***P*-value**

Petiole sap NO_3_^–^ and leaf tissue TN	Mid-July	0.15	0.02	0.64	0.58	0.34	0.05
	Late-July	0.29	0.09	0.36	0.54	0.29	0.07
	Mid-August	0.11	0.01	0.74	0.42	0.18	0.17
	Late-August	0.57	0.32	0.05	0.24	0.06	0.45

In the paired variable, the former variable (petiole sap NO_3_^–^) was treated as the independent variable (X) and the latter variable (leaf tissue TN) was treated as the dependent variable (Y) in linear regression analyses. Twelve paired samples were used for each Pearson correlation and linear regression analyses by nitrogen fertilizer rate treatments or sampling time in each year. Data were from a “Meeker” floricane raspberry field treated with different N fertilizer rates in Washington, United States.

Petiole sap K^+^ concentration was weakly negatively correlated to leaf tissue K concentration when pooled across 2 years (Figure 2). When the correlation of petiole sap K^+^ with leaf tissue K concentrations was compared by year, a weak and negative correlation between petiole sap K^+^ and leaf tissue K concentration was found in 2019 but the correlation was weak and positive in 2020 (Figure 2). No correlation between petiole sap K^+^ and leaf tissue K concentration was found for each sampling time in either year (Table 6).

**TABLE 6:** Pearson correlation coefficients (r), linear regression coefficients of determination (R^2^), and the *P*-values of paired variables by sampling time in 2019 and 2020.

Paired variable	Sampling time	2019			2020		
		Correlation coefficient (r)	Coefficient of determination (R^2^)	*P*-value	Correlation coefficient (r)	Coefficient of determination (R^2^)	*P*-value
Petiole sap K^+^ and leaf tissue K	Mid-July	0.31	0.10	0.32	0.36	0.13	0.25
	Late-July	0.01	0.00	0.97	0.08	0.01	0.81
	Mid-August	−0.54	0.29	0.07	−0.34	0.12	0.28
	Late-August	0.20	0.04	0.53	−0.23	0.05	0.47

In the paired variable, the former variable (petiole sap K^+^) was treated as the independent variable (X) and the latter variable (leaf tissue K) was treated as the dependent variable (Y) in linear regression analyses. Twelve paired samples were used for Pearson correlation and linear regression analyses by sampling time in each year. Data were from a “Meeker” floricane raspberry field treated with different N fertilizer rates in Washington, United States.

Petiole sap Ca^2+^ concentration was weakly positively correlated to leaf tissue Ca concentration when pooled across 2 years (Figure 2). When comparing the correlation of petiole sap Ca^2+^ and leaf tissue Ca concentrations by year, no correlation was found between petiole sap Ca^2+^ and leaf tissue Ca concentration in 2019, however, petiole sap Ca^2+^ and leaf tissue Ca concentration were weakly positively correlated in 2020 (Figure 2). In 2019, petiole sap Ca^2+^ and leaf tissue Ca concentration were moderately positively correlated in mid-July, and 42% of the variation in leaf tissue Ca concentration could be explained by the petiole sap Ca^2+^ concentration. No relationships between petiole sap Ca^2+^ and leaf tissue Ca concentration were observed in other sampling times in 2019 and 2020 (Table 7).

**TABLE 7:** Pearson correlation coefficients (r), linear regression coefficients of determination (R^2^), and *P*-values of paired variables by sampling time point in 2019 and 2020.

Paired variable	Sampling time	2019			2020		
		Correlation coefficient (r)	Coefficient of determination (R^2^)	*P*-value	Correlation coefficient (r)	Coefficient of determination (R^2^)	*P*-value
Petiole sap Ca^2+^ and leaf tissue Ca	Mid-July	0.65	0.42	0.02	0.49	0.24	0.10
	Late-July	−0.30	0.09	0.35	0.28	0.08	0.38
	Mid-August	0.09	0.01	0.77	0.08	0.01	0.80
	Late-August	0.22	0.05	0.50	0.01	0.00	0.98

In the paired variable, the former variable (petiole sap Ca^2+^) was treated as the independent variable (X) and the latter variable (leaf tissue Ca) was treated as the dependent variable (Y) in linear regression analyses. Twelve paired samples were used for Pearson correlation and linear regression analyses by sampling time in each year. Data were from a “Meeker” floricane raspberry field treated with different N fertilizer rates in Washington, United States.

### Relationship of petiole sap NO_3_^–^ and leaf tissue total nitrogen concentration with plant growth parameters, yield, and soil estimated nitrogen release

Pearson correlation and linear regression analyses of petiole sap NO_3_^–^ concentration, leaf tissue TN concentration with primocane height, primocane number, total fruit yield, and soil estimated nitrogen release were carried out with 2020 data (Table 8). Petiole sap NO_3_^–^ concentration had a similar trend as leaf tissue TN concentration when correlating to plant growth variables, total fruit yield, and soil estimated nitrogen release. Petiole sap NO_3_^–^ concentration was moderately positively correlated to primocane height and number (Table 8). The relationship between leaf tissue TN concentration with primocane height and number was both moderately positive (Table 8). However, both petiole sap NO_3_^–^ and leaf tissue TN concentrations had no correlation with total fruit yield nor soil estimated nitrogen release (Table 8).

**TABLE 8:** Pearson correlation coefficients (r), linear regression coefficients of determination (R^2^), and *P*-values of each paired variable in 2020.

Paired variable	Correlation coefficient (r)	Coefficient of determination (R^2^)	*P*-value	Paired variables	Correlation coefficient (r)	Coefficient of determination (R^2^)	*P*-value
Petiole sap NO_3_^–^ and primocane height (cm)	0.56	0.31	0.05	Leaf tissue TN and primocane height	0.68	0.46	0.01
Petiole sap NO_3_^–^ and primocane number	0.69	0.47	0.01	Leaf tissue TN and primocane number	0.63	0.40	0.03
Petiole sap NO_3_^–^ and yield^z^	−0.02	0.0005	0.95	Leaf tissue TN and yield	−0.51	0.26	0.09
Petiole sap NO_3_^–^ and soil ENR^y^	−0.47	0.22	0.12	Leaf tissue TN and soil ENR	−0.31	0.09	0.33

In each paired variable, the former variable (petiole sap NO_3_^–^ and leaf tissue TN) was treated as the independent variable (X) and the latter variable [primocane height, primocane number, soil estimated nitrogen release (ENR), and yield] was treated as the dependent variable (Y) in each linear regression analysis. Twelve paired samples were used for each Pearson correlation and linear regression analysis. Data were from a “Meeker” floricane raspberry field treated with different N fertilizer rates in Washington, United States.

^z^Total fruit yield of “Meeker” raspberry (kg plant^–1^) treated with different N fertilizer rates. Fruit were machine harvested every 3–4 days between 29 June and 4 August 2020.

^y^Soil estimated nitrogen release (ENR) was estimated based on the percentage of organic matter in the soil.

### Autocorrelation analysis

No significant autocorrelations were found in measured petiole sap nutrients and leaf tissue TN concentrations over sampling time in 2019 and 2020 (Table 9). Moderate negative autocorrelations were found both with leaf tissue K and Ca concentrations in 2020 when leaf samples were assayed for a longer period (lag 3). Thus, in 2020, leaf tissue K and Ca concentrations measured 6 weeks (lag 3) after the first measurement (mid-July) were related to the nutrient concentrations assayed in the first measurement, respectively (Table 9).

**TABLE 9:** Autocorrelation coefficients of measured “Meeker” floricane raspberry petiole sap and leaf tissue nutrient concentrations over time in 2019 and 2020 (lag 1: determination in 2 weeks; lag 2: determination in 4 weeks; lag 3: determination in 6 weeks).

Variable	Lag					
	2019			2020		
	1	2	3	1	2	3
Petiole sap NO_3_^–^	−0.20	−0.36	−0.29	−0.17	−0.25	−0.49
Leaf tissue N	−0.09	−0.13	−0.41	−0.34	0.12	−0.33
Petiole sap Ca^2+^	0.08	−0.24	−0.35	−0.13	−0.05	0.12
Leaf tissue Ca	0.03	−0.48	−0.12	−0.13	0.29	−0.71*
Petiole sap K^+^	−0.03	−0.32	−0.03	0.11	0.003	−0.40
Leaf tissue K	−0.16	0.20	−0.26	0.03	0.22	−0.56*

The nutrient concentration in the following 2, 4, and 6 weeks was estimated based on the nutrient concentration observed 2, 4, and 6 weeks ago, respectively.

*Indicates significant differences at *P* ≤ 0.05.

### Costs of plant nutrient tests

Raspberry growers are recommended to annually assess plant nutrient concentrations to guide nutrient management practices (Hart et al., 2006). Petiole sap and conventional leaf tissue nutrient analyses will require 25 and 5 leaves, respectively, according to the section “Materials and methods” in the current study. Therefore, approximately 30 leaf samples are needed each year for nutrient analysis using both testing methods. For conventional tissue nutrient analyses, the labor cost for collecting 30 plant samples is $45 ($15 person^–1^ hr^–1^, benefits included; Galinato and DeVetter, 2016). The cost for laboratory analyses of the 30 samples is $1,050 ($35 sample^–1^) plus a $50 packing and shipping fee. Thus, a Washington red raspberry grower would pay approximately $1,145 each year for this sample analysis method. For the rapid sap analyses with compact ion meters, assuming that three ion selective electrode meters are needed to measure sap NO_3_^–^, K^+^, and Ca^2+^ concentrations, the fixed purchasing cost is approximately $1,120 for all three meters (Horiba Inc., Irvine, CA, United States). Additionally, the cost for a plant press and pruner for sample collection is $100, and the cost of additional supplies (e.g., Kimtech wipe, pipette, paper bag, calibration solution, battery) is approximately $100 per year. The labor cost is $135 for both sampling and testing the 30 samples at $15 person^–1^ hr^–1^ (rate includes benefits; Galinato and DeVetter, 2016). Another cost consideration for the rapid sap test is the sensors should be replaced every 2 years, at an average additional cost of $450 for each replacement of all three meters whereas the body of the meter can last for many years if well maintained (Samuel Francois from HORIBA instruments company, personal communication). Thus, the total cost of sap analyses with compact ion meters will be approximately $1,455 each year for the first 2 years but only $460 per year in the following years.

For the 6-year expected lifespan of a red raspberry planting in Washington, the total cost of conventional tissue nutrient analysis would be $6,870 whereas the cost of sap analysis with compact ion meters would be $3,530. Thus, sap analysis would provide an estimated $3,340 cost savings over conventional tissue nutrient analysis. More cost savings are anticipated with sap analysis in the long run and for large quantities of samples. However, it is important to note that the cost of sap analysis would increase with the purchase of additional compact ion meters to analyze each additional nutrient, whereas the cost of conventional tissue nutrient analysis would remain the same. Further, sap analysis tests are only possible for a limited number of nutrients, NO_3_^–^, K^+^, Ca^2+^, and Na^+^ (Samuel Francois from HORIBA instruments company, personal communication), whereas conventional tissue nutrient analysis is available for all macro- and micro-nutrients. An additional advantage of compact ion meters is that they could also be used to test soil nutrients (Horiba Inc., Irvine, CA, United States).

## Discussion

All measured petiole sap and leaf tissue nutrient concentrations did not vary by N fertilizer rate. Plant N status was considered adequate across all N fertilizer rates (including the unfertilized control) given that measured leaf tissue TN concentrations were within the sufficiency range of 2.3–3.0% proposed by Hart et al. (2006). This range is for cranberries produced in western Oregon, United States, which has a similar production environment as northwest Washington where this study was carried out.

The lack of a petiole sap NO_3_^–^ and leaf tissue TN response to fertilizer rate was unexpected. In this study, fertilized and unfertilized soil had similar soil organic matter content and estimated nitrogen release (Supplementary Table 5), which may partially explain the lack of plant response to the N fertilizer rate. Soil estimated nitrogen release in this study was 98.6–100.5 kg N ha^–1^, which exceeded the recommended annual N fertilizer rate (56–90 kg N ha^–1^) for raspberry production in Washington (Supplementary Table 5; Hart et al., 2006; DeVetter et al., 2022). Utilization of nutrients stored in plant reserves may also impede plant response to the N fertilizer rate. It has been reported that floricane red raspberry utilizes approximately 40% of its stored N per year to fuel cane, crown, and root growth (Rempel et al., 2004), which may also limit observations of a clear N fertilizer rate effect. The lack of plant response to N fertilizer rate in this study was in line with Rizzi et al. (2020) who found that leaf tissue TN concentration of “Autumn Bliss” primocane red raspberry cultivated in southern Brazil did not differ by N fertilizer rate.

Raspberry is a perennial plant and a year effect found in the current study was expected due to plant aging and variations in annual weather (Figure 1). Year effects were also observed in “Meeker” raspberry and “Thornfree” trailing blackberry (*Rubus ursinus* Cham. and Schldl.) treated with different fertilizer regimes in western Serbia (Milošević et al., 2018; Stojanov et al., 2019). Petiole sap NO_3_^–^ concentration of cotton cultivated in Tennessee, United States (Zhou and Yin, 2018) and leaf tissue TN concentration of “Willamette” floricane red raspberry cultivated in British Columbia, Canada (Kowalenko, 1994) differed significantly across sampling time, which was in line with the findings in the current study that significant time effects were observed for both petiole sap NO_3_^–^ and leaf tissue TN concentrations. The cranberry nutrient management guide from western Oregon recommends collecting leaf tissue samples from late-July to early-August as leaf tissue N status is relatively stable during this period (Hart et al., 2006). Outside of these time periods, raspberry leaf tissue TN concentration change rapidly early in the growing season as nutrients are translocated to vegetative canes, flowers, and fruits and decreases dramatically when plants begin to acclimate for winter and leaves senesce as nutrients are translocated to roots, crown, and over-wintered canes (Rempel et al., 2004; Strik and Bryla, 2015). The current research findings indicated that the right sampling time is critical for petiole sap and leaf tissue analysis.

Plant K status was considered adequate given all mean values of leaf tissue K concentrations were within the range considered sufficient for cranberries (1.3–2.0%) (Hart et al., 2006). Plants in the current study were also sufficient in Ca given that all mean values of leaf tissue Ca concentrations were within or slightly below the sufficiency range (0.6–2.0%) for cranberry (Hart et al., 2006). Baseline soil levels also indicated K and Ca were not limited according to the grading rule created by A&L Western Agricultural Laboratories (Table 1; A&L Western Laboratories, Inc., Portland, OR, United States).

The non-significant correlation between petiole sap NO_3_^–^ and leaf tissue TN concentration found at some sampling times in this study was in line with Carson et al. (2016) who found no correlation between tomato petiole sap NO_3_^–^ and leaf tissue TN concentration at the first bud, first open flower, second harvest, and third harvest stages. However, a weak positive correlation (*r* = 0.38) was found between tomato petiole sap NO_3_^–^ and leaf tissue TN concentration across 2 years (Carson et al., 2016), which was in contrast to the insignificant correlation found in the current study.

The insignificant correlation found in the current study may be due to physiological, environmental, and methodological factors. Concerning physiological factors, NO_3_^–^ is a mobile nutrient in plants and is only part of the leaf tissue TN, which includes nucleic acids, amino acids, proteins, chlorophyll, ammonium, and other compounds. NO_3_^–^ in petiole sap can be absorbed, transported, metabolized, assimilated, and thereby changes into other organic and inorganic forms of N, and these inherent physiological changes of NO_3_^–^ in petiole sap may not be correspondingly reflected in leaf tissue TN (Taiz et al., 2015). In addition, raspberry can absorb ammonium released from urea fertilizer through urease degradation in soil, which is then converted to other forms of N in the plant, thus, absorbed ammonium could increase leaf tissue TN concentration but not petiole sap NO_3_^–^ concentration (Carson et al., 2016).

With respect to environmental factors, Llanderal et al. (2019, 2020) speculated that tomato petiole sap NO_3_^–^ concentration could be impacted by air temperature, relative humidity, and global radiation. These environmental factors varied day to day from mid-July to late-August in western Washington with its Mediterranean climate (Figure 1), which might contribute to the insignificant correlation of petiole sap NO_3_^–^ and leaf tissue TN concentrations. Moreover, petiole sap NO_3_^–^ and leaf tissue TN concentrations had maximum and minimum values at different sampling times. These variations by sampling time could also contribute to the lack of correlations.

Regarding methodological factors, petiole sap NO_3_^–^ concentration was measured using a compact meter with an ion-selective electrode. Ion interference caused by other organic or inorganic ions in sap might partially contribute to the lack of correlations between petiole sap NO_3_^–^ and leaf tissue TN concentration in this study (Folegatti et al., 2005; Rundle, 2011). Additionally, sap NO_3_^–^ concentration was measured only with petioles whereas TN concentration was measured with the whole leaf tissue (including the petiole, leaflet, and petiolule). This difference in analytical samples may contribute to the lack of correlation between petiole sap NO_3_^–^ and leaf tissue TN concentration. Nitrogen taken up during peak uptake is mainly assimilated or stored in vegetative tissues and has not been translocated to other plant tissues; therefore a strong correlation between petiole sap NO_3_^–^ and leaf tissue TN is often expected during this growth stage (Caldwell et al., 2010; Shi et al., 2019). Nitrate stored in leaf vacuoles starts translocating to flowers and fruits on spatially separated floricanes during the reproductive growth stage and to root, crown, and over-wintered primocanes during the end of the growing season (Rempel et al., 2004; Taiz et al., 2015). Previous research has indicated uptake of nitrogen in primocanes continues through August but varies with fertilizer application time in terms of where it is allocated among floricane and primocane tissues, which further complicates selecting the right sampling timing. In addition, nitrogen can come from stored tissues. All these dynamics may contribute to the non-strong correlations between petiole sap NO_3_^–^ and leaf tissue TN concentrations found in the current study (Rempel et al., 2004). Utilization of new leaves (the first most recently fully expanded leaf) for both mobile and immobile nutrients may have been a limitation as well and analyzing other organs may be explored in future studies.

The negative relationship between petiole sap K^+^ and leaf tissue K concentration found in this study across 2 years contrasts with the results reported by Kallenbach (2000) and Noguchi et al. (2005), who found a positive correlation between alfalfa (*Medicago sativa* L.) leaf sap K^+^ and leaf tissue K concentration and between luffa (*Luffa cylindrica* Roem.) xylem sap K^+^ and shoot tissue K concentration, respectively. Moreover, Gangaiah et al. (2016) reported strong positive correlations between diluted petiole plus midrib sap K^+^ and dried tissue K concentration in pak choi fertilized with different K fertilizer rates. There are some potential limitations to exploring the relationship between petiole sap K^+^ and leaf tissue K concentration due to the small ranges in petiole sap K^+^ and leaf tissue K concentrations in this study. The weak and inconsistent relationship for K in the current study may be due to the physiological, environmental, and methodological factors. Potassium is a mobile nutrient that can undergo luxury uptake and is physiologically important to establish cell turgor and maintain cell electroneutrality (Taiz et al., 2015). The high mobility of K and luxury uptake likely contributes to the weak and inconsistent relationship between petiole sap K^+^ and leaf tissue K concentration observed in this study. Additionally, differing trends of sampling time effects for petiole sap K^+^ and leaf tissue K concentrations indicate the plant growth stage and environment may affect the relationship between petiole sap K^+^ and leaf tissue K concentrations. With respect to methodological factors, the interference of other ions in the sap might impact K^+^ readings in the compact ion meter (Folegatti et al., 2005; Di Gioia et al., 2010; Parks et al., 2012). In addition, only ionic K^+^ from petiole sap was measured using the compact ion meter, whereas all forms of K (enzyme cofactor K included) from the whole leaf tissue were measured as leaf tissue K concentration.

The weak positive correlation between petiole sap Ca^2+^ and leaf tissue Ca concentration in this study was in contrast with Bundt et al. (1997) and Noguchi et al. (2005), who reported a moderate positive correlation between luffa xylem sap Ca^2+^ and shoot tissue Ca concentration and a strong correlation between coffee (*Coffea arabica* L.) xylem sap Ca^2+^ and leaf tissue Ca concentration, respectively. Given that no Ca fertilizers were applied in the current study, there are some potential limitations to exploring the relationship between petiole sap Ca^2+^ and leaf tissue Ca concentration due to the small ranges of the concentrations. Ca^2+^ is an immobile nutrient and a constituent of leaf tissue Ca. Leaf tissue Ca includes Ca present in the middle lamella and cell walls, enzyme cofactors, and secondary messengers (Taiz et al., 2015). Therefore, petiole sap Ca^2+^ is not representative of all the Ca in whole leaf tissues, which also included the petiole, leaflet, and petiolule (Gangaiah et al., 2016). Petiole sap Ca^2+^ can also be absorbed, metabolized, assimilated, and thereby form other compounds containing Ca, however, these changes would not reflect in leaf tissue Ca. Petiole sap Ca^2+^ and leaf tissue Ca concentrations also had different trends across sampling time, which could be impacted by the environment (White and Broadley, 2003). Detection of Ca^2+^ concentration with a compact ion meter could be interfered by the presence of other ions in the sap (Di Gioia et al., 2010; Parks et al., 2012). These combined factors can contribute to the weak relationship between petiole sap Ca^2+^ and leaf tissue Ca concentration.

Both petiole sap NO_3_^–^ and leaf tissue TN concentration were moderately positively correlated to primocane height and primocane number, which was within expectation as N nutrition is closely associated with raspberry growth (Rempel et al., 2004; Hart et al., 2006). The insignificant correlation of petiole sap NO_3_^–^ and leaf tissue TN concentration with yield in this study was in contrast with Majić et al. (2008), who found both petiole sap NO_3_^–^ and leaf tissue TN concentration was positively correlated to potato tuber yield, and with Carson et al. (2016) who found a positive correlation between leaf tissue TN concentration and marketable tomato yield but a negative relationship between petiole sap NO_3_^–^ concentration and marketable tomato yield. The non-significant relationship of both petiole sap NO_3_^–^ and leaf tissue TN concentration with yield was likely due to the lack of plant response to N fertilizer rates (Caldwell et al., 2010). Correlations of petiole sap NO_3_^–^ and leaf tissue TN concentration with soil estimated nitrogen release were also lacking. This likely accounted for the lack of plant response to N fertilizer rates as plants might accumulate sufficient N from plant nutrient reserves and mineralization of soil organic matter to meet plant needs in all N fertilizer rate treatment plots (Rempel et al., 2004; Strik and Bryla, 2015).

Results of the autocorrelation reflected the rapid and random variation of the measured petiole sap and leaf tissue nutrient concentrations throughout the sampling time, which further validated the power of established linear regression techniques to effectively capture time trends. The non-significant autocorrelation over the sampling time might be attributed to the prompt plant self-regulation of accumulated ions in the vacuole of storage tissues. The significant autocorrelation over the sampling time for both leaf tissue K and Ca concentrations reflected the limited ability of plants to regulate the corresponding nutrient concentrations in response to biotic and abiotic impacts in the sampled plant organ during the sampling period (Llanderal et al., 2020).

There were significant expected cost savings with sap analysis compared to conventional tissue nutrient analysis over the 6-year expected lifespan of a red raspberry planting. Based on the success of sap analysis for plant nutrient assessment in other crops, further research is warranted to investigate the right sampling time and plant organs under various N, K, and Ca sufficiency levels to study if this method can be used to predict plant nutrient status and application needs in red raspberry.

## Conclusion

Raspberry petiole sap NO_3_^–^, K^+^, Ca^2+^ and leaf tissue TN, K, and Ca concentrations did not differ by N fertilizer rate treatments but differed by year and sampling time depending on the nutrient. Due to the lack of plant response to N fertilizer rate and non-strong and inconsistent correlations between petiole sap NO_3_^–^ and leaf tissue TN concentration, petiole sap K^+^ and leaf tissue K concentration, and petiole sap Ca^2+^ and leaf tissue Ca concentration, results from this study were inconclusive. Our results were suggestive that developing robust nutrient management plans on the basis of petiole sap for N, K, and Ca status in floricane raspberry would be challenging. Based on the cost savings and the shorter time for attaining results with the sap analysis compared to conventional tissue nutrient analysis, growers’ interest in rapid sap analysis might increase. Thus, further field research to investigate the right sampling time and plant organs under various N, K, and Ca sufficiency levels to study the feasibility of using plant sap analysis to guide real-time decision-making may be valuable.

## Data availability statement

The raw data supporting the conclusions of this article will be made available by the authors, without undue reservation. Environmental data is available for free via WSU AgWeatherNet. All other data are available upon request to the corresponding authors.

## Author contributions

QL: conceptualization, methodology, software, validation, formal analysis, investigation, data curation, writing – original draft and review and editing, and visualization. CM: conceptualization, methodology, resources, and writing – review and editing. HT: resources, writing – review and editing, and funding acquisition. LD: conceptualization, methodology, resources, writing – review and editing, project administration, and funding acquisition. All authors contributed to the article and approved the submitted version.
