# Supplementary material for: Evaluation of real-time nutrient analysis of fertilized raspberry using petiole sap
Source: Front Plant Sci. 2022 Aug 5;13:918021. doi: 10.3389/fpls.2022.918021 (PMC9389145; doi:10.3389/fpls.2022.918021)
Supplement: Supplementary file 1 [file Data_Sheet_1.docx]

Supplementary Table S1. Significance (displayed as *P*-values) of nitrogen fertilizer rate treatments, year, and treatment by year interactions for measured floricane raspberry petiole sap and leaf tissue nutrients concentrations throughout 2019 and 2020. The significance of total fruit yield, primocane height, primocane number, soil organic matter (OM), soil estimated nitrogen release (ENR), soil potassium (K), and soil calcium (Ca) measured in 2020 are also displayed.

| Variable | Treatment | Year | Treatment*year |
| --- | --- | --- | --- |
| Petiole sap NO_3_^-^ | 0.95 | 0.59 | 0.38 |
| Leaf tissue N | 0.65 | 0.11 | 0.70 |
| Petiole sap Ca^2+^ | 0.89 | 0.11 | 0.63 |
| Leaf tissue Ca | 0.59 | 0.005 | 0.43 |
| Petiole sap K^+^ | 0.53 | 0.0002 | 0.59 |
| Leaf tissue K | 0.91 | <0.0001 | 0.58 |
| Fruit yield | 0.85 | - ^z^ | - |
| Primocane height | 0.72 | - | - |
| Primocane number | 0.11 | - | - |
| Soil OM | 0.75 | - | - |
| Soil ENR | 0.86 | - | - |
| Soil K | 0.57 | - | - |
| Soil Ca | 0.06 | - | - |

^z^ (-) Not applicable as these variables were only measured in 2020.

Supplementary Table S2. ‘Meeker’ floricane red raspberry petiole sap potassium (K^+^), leaf tissue K and calcium (Ca) concentrations by year given significant year effects.

| Year | Petiole sap K^+^ (ppm) | Leaf tissue K (%) | Leaf tissue Ca (%) |
| --- | --- | --- | --- |
| 2019 | 4738.54 ± 100.73 a ^z^ | 1.62 ± 0.04 b | 0.65 ± 0.0007 a |
| 2020 | 4207.29 ± 88.23 b | 1.86 ± 0.03 a | 0.61 ± 0.01 b |
| *P*-value | 0.0002 | <0.0001 | 0.005 |

^z^ Data are displayed as means ± SE (standard error) (n = 48); means followed by different letter within a column are significantly different at P ≤ 0.05 using a means comparison with a Tukey’s honestly significant difference test.

Supplementary Table S3. Significance (displayed as *P*-values) of nitrogen fertilizer rate treatments, sampling time, and their interactions for measured ‘Meeker’ floricane red raspberry petiole sap and leaf tissue nutrient concentrations, 2019 and 2020.

| Variable | 2019 | | |  | 2020 | | |
| --- | --- | --- | --- | --- | --- | --- | --- |
|  | Treatment | Sampling time | Treatment*sampling time |  | Treatment | Sampling time | Treatment*sampling time |
| Petiole sap NO_3_^-^ | 0.68 | <0.0001 | 0.20 |  | 0.94 | <0.0001 | 0.88 |
| Leaf tissue N | 0.47 | 0.003 | 0.23 |  | 0.79 | 0.09 | 0.96 |
| Petiole sap Ca^2+^ | 0.83 | 0.0003 | 0.16 |  | 0.49 | 0.006 | 0.94 |
| Leaf tissue Ca | 0.68 | 0.0001 | 0.43 |  | 0.48 | 0.008 | 0.89 |
| Petiole sap K^+^ | 0.29 | <0.0001 | 0.75 |  | 0.39 | <0.0001 | 0.46 |
| Leaf tissue K | 0.57 | 0.0007 | 0.75 |  | 0.76 | 0.001 | 0.60 |

Supplementary Table S4. Significance (displayed as *P*-values) of N fertilizer rate treatments within each sampling time on measured ‘Meeker’ floricane red raspberry petiole sap and leaf tissue nutrient concentrations, 2019 and 2020.

| Variable | 2019 | | | |  | 2020 | | | |
| --- | --- | --- | --- | --- | --- | --- | --- | --- | --- |
|  | Mid-July | Late-July | Mid-August | Late-August |  | Mid-July | Late-July | Mid-August | Late-August |
| Petiole sap NO_3_^-^ | 0.99 | 0.87 | 0.06 | 0.63 |  | 0.90 | 0.67 | 0.90 | 0.62 |
| Leaf tissue N | 0.41 | 0.26 | 0.68 | 0.11 |  | 0.67 | 0.99 | 0.81 | 0.97 |
| Petiole sap Ca^2+^ | 0.18 | 0.99 | 0.19 | 0.22 |  | 0.75 | 0.27 | 0.35 | 0.99 |
| Leaf tissue Ca | 0.20 | 0.23 | 0.43 | 0.86 |  | 0.38 | 0.61 | 0.86 | 0.52 |
| Petiole sap K^+^ | 0.44 | 0.10 | 0.88 | 0.68 |  | 0.35 | 0.46 | 0.36 | 0.60 |
| Leaf tissue K | 0.92 | 0.39 | 0.59 | 0.89 |  | 0.88 | 0.24 | 0.88 | 0.19 |

Supplementary Table S5. Soil organic matter (SOM), estimated nitrogen release (ENR), potassium (K), and calcium (Ca) concentrations by nitrogen fertilizer rate treatments in 2020.

| Treatment | | SOM (%) | ENR ^y^ (kg N ha^-1^) | K (ppm) | Ca (ppm) |
| --- | --- | --- | --- | --- | --- |
| Control (0 kg N ha^-1^) | 3.80 ± 0.13 ^z^ | | 98.6 ± 1.7 | 250.7 ± 6.2 | 1445.7 ± 25.8 |
| Low (34 kg N ha^-1^) | 3.87 ± 0.18 | | 99.8 ± 1.9 | 211.3 ± 5.2 | 1333.3 ± 10.8 |
| Medium (67 kg N ha^-1^) | 4.00 ± 0.12 | | 100.5 ± 1.4 | 232.3 ± 12.9 | 1425.0 ± 59.4 |
| High (101 kg N ha^-1^) | 3.95 ± 0.12 | | 100.1 ± 1.5 | 228.0 ± 13.0 | 1458.7 ± 50.0 |
| *P*-value | 0.75 | | 0.86 | 0.57 | 0.06 |

^z^ Data are displayed as means ± SE (standard error) (n = 3); means followed by different letter within a column are significantly different at *P* ≤ 0.05 using a means comparison with a Tukey’s honestly significant difference test.

^y^ Soil ENR was estimated based on the percentage of organic matter in the soil.
